# Shared and genetically distinct *Zea mays* transcriptome responses to ongoing and past low temperature exposure

**DOI:** 10.1186/s12864-018-5134-7

**Published:** 2018-10-20

**Authors:** Luis M Avila, Wisam Obeidat, Hugh Earl, Xiaomu Niu, William Hargreaves, Lewis Lukens

**Affiliations:** 1 Department of Plant Agriculture, University of Guelph, 50 Stone Road East, Guelph, ON N1G 2W1 Canada; 2 Dupont/Pioneer, 7300 NW 62nd Ave, DuPont Pioneer, Johnston, Iowa, 50131 USA

**Keywords:** Maize, Cold, Abiotic stress, RNA-Seq, Short read alignment, Genotype environment interaction, Crossover interactions

## Abstract

**Background:**

Cold temperatures and their alleviation affect many plant traits including the abundance of protein coding gene transcripts. Transcript level changes that occur in response to cold temperatures and their alleviation are shared or vary across genotypes. In this study we identify individual transcripts and groups of functionally related transcripts that consistently respond to cold and its alleviation. Genes that respond differently to temperature changes across genotypes may have limited functional importance. We investigate if these genes share functions, and if their genotype-specific gene expression levels change in magnitude or rank across temperatures.

**Results:**

We estimate transcript abundances from over 22,000 genes in two unrelated *Zea mays* inbred lines during and after cold temperature exposure. Genotype and temperature contribute to many genes’ abundances. Past cold exposure affects many fewer genes. Genes up-regulated in cold encode many cytokinin glucoside biosynthesis enzymes, transcription factors, signalling molecules, and proteins involved in diverse environmental responses. After cold exposure, protease inhibitors and cuticular wax genes are newly up-regulated, and environmentally responsive genes continue to be up-regulated. Genes down-regulated in response to cold include many photosynthesis, translation, and DNA replication associated genes. After cold exposure, DNA replication and translation genes are still preferentially downregulated. Lignin and suberin biosynthesis are newly down-regulated. DNA replication, reactive oxygen species response, and anthocyanin biosynthesis genes have strong, genotype-specific temperature responses. The ranks of genotypes’ transcript abundances often change across temperatures.

**Conclusions:**

We report a large, core transcriptome response to cold and the alleviation of cold. In cold, many of the core suite of genes are up or downregulated to control plant growth and photosynthesis and limit cellular damage. In recovery, core responses are in part to prepare for future stress. Functionally related genes are consistently and greatly up-regulated in a single genotype in response to cold or its alleviation, suggesting positive selection has driven genotype-specific temperature responses in maize.

**Electronic supplementary material:**

The online version of this article (10.1186/s12864-018-5134-7) contains supplementary material, which is available to authorized users.

## Background

Chilling and cold temperatures cause numerous changes in plant biochemistry, development, and physiology [1]. Chilling and cold have immediate effects on membrane fluidity and enzyme kinetics, thereby affecting numerous cellular processes. They also affect transcript and protein abundances that have longer term effects [2, 3].

Maize has long been a model species to investigate cold responses and undergoes numerous changes in response to cold temperatures. In plants exposed to cold temperatures (e.g. temperatures lower than 5 °C but greater than 0 °C), plant growth ceases and leaves yellow [4]. Leaves become water deficient [5, 6] due to reduced hydraulic conductivity of the roots [7]. Photosynthesis is reduced or does not occur [8]. Levels of reactive oxygen species (ROS) increase [9–11] as do levels of ABA [6] and lipid peroxidation [12]. Activities of ROS responsive enzymes increase [12, 13] and lutein, neoaxanthin, and xanthophyll cycle carotenoids increase relative to chlorophyll a and b [14].

Because of their breadth, transcriptome analyses can identify the underlying biological processes that differ between samples [15]. A general principle is that one can infer how samples differ by determining if genes that differ between samples are involved in a common biological process or share molecular functions.

In maize, cold affects transcript abundance of many genes. A whole genome expression study using a microarray with 46,128 probes reports 2449 genes are responsive to cold in two maize inbred lines [16]. A more recent study of three inbred lines reports that over 30% of the expressed genes (~ 7000 genes out of ~ 20,000 expressed genes) are differentially expressed in cold relative to control conditions in each genotype [17]. In maize, functional annotations “protein phosphorylation” and “transcription” are overrepresented in genes induced by low temperatures, and the “photosythesis” term is overrrepresented in genes repressed by low temperatures [16].

Growth after the alleviation of chilling (12–15 °C) and cold temperatures (3–7 °C) also induces numerous cellular changes. Maize exposed to cold temperatures and then to warmer temperatures may appear bleached [8]. Necrotic leaf areas, covering almost entire leaves, may appear several days after the cold stress, and plants may die [6, 8]. Exposure to chilling temperatures (and low water availability) also induces processes that are active after chilling exposure and cause tolerance to future chilling temperature exposure [6, 18–21]. Chilling pretreatment reduces leaf necrosis and increases photosynthesis relative to untreated plants.

Multiple studies have shown that the transcriptomes of plants recovering from cold or other stresses mostly resemble the transcriptomes of plants never exposed to the stresses [22, 23]. Nonetheless, transcriptomes do change in response to past stress exposure. For example, Coolen et al. [23] exposed *Arabidopsis thaliana* to three different stresses individually and sequentially. Two plants exposed to the same stress have similar transcriptomes. However, if one plant was first exposed to a different stress, its transcriptome has detectable elements of this first stress treatment. Gene regulatory changes caused by past stress exposure may be due to the maintenance of genes differentially expressed during the stress. Alternatively, a set of genes may come under novel regulation because of the stress alleviation. In addition, genes differentially expressed after a stress may be adaptive, contributing to stress tolerance or be symptomatic of stress damage that occurred as a result of stress exposure.

Like other species, maize harbors genetic diversity both for physiological and developmental responses to chilling, cold and to their alleviation [4, 24, 25]. For example, under cold field conditions, Verheul et al. [25] found that three genotypes bred for temperate environments grew more quickly and had higher rates of net photosynthesis than three genotypes bred for tropical environments. While grown in cool temperatures, chilling tolerant genotypes maintain higher rates of photosynthesis and chlorophyll content relative to chilling intolerant genotypes [26]. Chilling tolerant genotypes have higher concentrations of β-carotene, lower concentrations of xanthophyll carotenoids, and a reduced proportion of zeaxanthin in their xanthophyll cycle carotenoid pool than do chilling susceptible genotypes [14, 27]. ABA accumulation in tolerant inbred lines is triggered at a higher leaf water potential than in less tolerant lines, and ABA reaches higher levels in the tolerant lines [6]. Following chilling or cold exposure, genotypes also vary for growth [6, 8]. Some lines are better than others at recovering photosynthesis capacity [8, 26, 28], and susceptible lines have great increases in oxidative damage after growth in cold relative to more tolerant lines [8].

Given the genetic variability of biochemical and developmental traits among cold grown and cold recovering plants, one may expect that genotypes’ transcriptome responses to cold and to cold recovery vary. Investigations suggest this expectation is largely correct, although more transcripts changed in all genotypes in response to temperature than in only a subset of genotypes. A microarray study of two maize inbred lines of contrasting cold sensitivity reported 66 genes with significantly different response to cold in one inbred relative to the other [16]. 2449 genes responded to cold temperatures in a similar manner in the two lines (*P* < 0.1). RNA-Seq measures transcript abundances more accurately than do microarrays, although estimates of different genotypes’ expression may be affected by sample and reference genome polymorphisms. Within three sets of inbred pairs, Waters et al. [29] identified 308, 584, and 8075 genes with significant genotype x temperature interactions using RNA-Seq. Many genes also have genotype specific responses to cold recovery in rice [22, 30, 31].

Genotype-specific transcriptome responses may suggest genotype-specific trait responses. For example, the 66 genes having genotype-specific cold temperature responses in Sobkowiak et al. [16] are enriched for carbohydrate and amino acid metabolism, signal transduction pathways, and redox potential homeostasis. However, the connection between genotype-specific transcriptome responses and genotype-specific trait responses can be difficult to elucidate. Much gene expression variation that is induced by environmental variation is thought to be maladaptive or neutral [32]. Non-essential genes and genes under complex regulatory control are more likely to have genotype- specific environmental responses than other genes [33]. Two factors would suggest that gene expression changes explain phenotypic variation. First, genes that change may be clustered within functional groups that are known to affect a phenotype. Second, transcripts within a pathway or process may be consistently up- or down- regulated in one genotype relative to another.

In this study, we investigate how two unrelated maize genotypes respond to cold temperatures and to the alleviation of cold temperatures using RNA-Seq. Genotype consistently and cold treatment temporarily affect the global similarity of maize transcriptomes. Partitioning gene expression variation to additive and interaction effects identifies novel, diverse cold- and recovery-responsive genes and pathways. Results suggest that a number of biochemical responses to cold and its alleviation are under transcriptional control. Genotype-specific responses to cold and the alleviation of cold are enriched for specific processes. These processes are strongly differentiated between genotypes suggesting the functional importance of genotype x environment interactions.

## Methods

### Plant growth conditions

Maize seedlings of genotypes CG60 and CG102 were grown in growth chambers for 13 days at 24 °C/14 °C day/night temperature, with a 16-h photoperiod and relative humidity of approximately 60/70% (day/night). CG60 and CG102 are adopted to short season growing regions yet are from different breeding pools. They are parents of high-yielding inbred lines, and are parents of a RIL population with a published genetic map [34, 35]. Plants were grown in a 1:1 (*v*/v) mixture of fine (“Quick Dry”) Turface (Profile Products, Buffalo Grove IL) and Promix PGX (Premiere Tech, Rivere-du-Loup QC) and were supplied daily with a nutrient solution composed of 164 g 20–20-20 N-P-K, 75 g Ca(NO_3_)_2_.H_2_O, 128 g MgSO_4_.7H_2_O, 64 g NH_4_NO_3_ and 9.6 g Micronutrient Mix (Plant Products, Brampton ON) dissolved in 20 L of tap water. The nutrient solution was applied with the irrigation water through a 1:20 in-line mixer. The photosynthetic photon flux density during the photoperiod was maintained at 650 μmol m^− 2^ s^− 1^ at the top of the canopy, with a mixture of “cool white” fluorescence tubes and “inside frost” tungsten bulbs (Osram Sylvania, Drummondville, QC). For both the control and stress chambers we used a ramp type temperature protocol, such that the daily high temperature was maintained from noon until 4 pm, then the temperature declined in a linear fashion from 4 pm until 1 am. The daily low temperature was maintained from 1 am until 5 am, and then the temperature increased in a linear fashion from 5 am until noon.

**Fig. 1:**
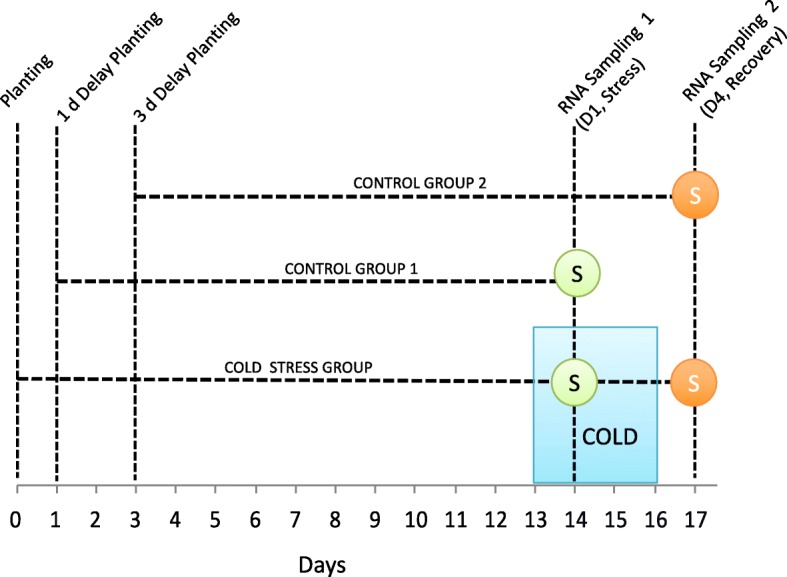
Experimental design. All plants were first germinated and grown at 24 °C /14 °C day/ night temperature. The cold treated plants were exposed to 14 °C / 2 °C day/night temperatures for three days starting on day 13 after planting and then returned to 24 °C /14 °C. Leaf samples for RNA extraction, depicted by the letter S, were taken 24 h into the cold exposure (D1) and 24 h after cold exposure (D4). Two control groups of plants were planted with one and three day delays, so the developmental stages of the cold and control treated plants were the same at sampling time

At the second leaf stage (V2, two leaves with visible ligules, 13 days after planting, circa 0.1 g dry weight), plants to receive the cold stress treatment were transferred to 14 °C/2 °C for 3 days. They then recovered at 24 °C/14 °C. We sampled cold-treated plants 24 h into the cold stress (14 days after planting, first day of sampling, D1) and 24 h into the recovery period (17 days after planting, second day of sampling, D4). For each sampling time we had control plants grown under the 24 °C/14 °C day/night temperature regime for all their development. Knowing that low temperatures slow down development, in an attempt to match the development stage of control and stressed plants we planted the control plants 1 and 3 days after the cold stress treated plants were planted, so that at time of tissue collection the control plants were 13 (D1) and 14 (D4) days old (Fig. 1). The experiment was repeated three times. Cold grown plants harvested 3 days after the end of cold exposure, had approximately one-half the aboveground biomass of control-grown plants. In the cold treatment, plants were sampled for RNA extraction at 14 °C.

Cold tolerance is defined as the ability to maintain biomass accumulation in cold temperatures. The ratios of cold treated plant / control plant dry weights were calculated for both inbreds with seven replicates in time. The mean biomass stress: control ratio of CG60 (0.54) was significantly higher than the mean ratio of CG102 (0.47; t-test, *p* < 0.02). We also measured two spectral reflectance indices, the photochemical reflectance index, PRI and the normalized difference red edge index, NDRE. NDRE is the reflectance at 790 nm minus the reflectance at 720 nm divided by the sum of the two, and NDRE correlates with leaf greenness (e.g. [34]). PRI is the reflectance at 531 nm minus the reflectance of 570 nm divided by the sum. PRI negatively correlates both with the ratio of carotenoids to chlorophyll [36] and with the epoxidation state of xanthophyll cycle carotenoids [37]. Leaf reflectance was measured at 3.3-nm resolution using a reflectance spectrometer (UniSpec-SC Spectral Analysis System, PP Systems, Amesbury MA). PRI decreased in both CG60 and CG102 due to cold stress, but the change was not significant (*P* < 0.06). PRI decreased more in CG102 than CG60 due to cold, but the difference was also not significant (*P* < 0.32). NDRE declined in both genotypes in response to cold (*P* < 0.03). The decrease was greater in CG102 than CG60 (the ratio of cold exposed to control NDRE is 0.48 and 0.53, respectively), but the difference was not significant (*P* = 0.15). Finally, using a miniPam chlorophyll fluorometer (Heinz Walz GmbH, Effeltrich, Germany) we measured the maximum efficiency of photosystem II (Fv/Fm) in leaves that had been dark-adapted for 30 min. Cold significantly reduced Fv/Fm relative to control plants (*P* < 0.04). In addition, CG60 was reduced significantly less than CG102 (*P* < 0.01). In sum, the biomass, spectral, and fluorometry measurements indicate CG60 is more cold tolerant than CG102.

### RNA extraction, pooling and sequencing

For each genotype x treatment x day of sampling combination, the second leaves from the base of each of five plants were harvested and pooled together to generate a single replicate. The second leaf is the second leaf counting up from the soil surface not including the coleoptile. The whole leaf blade was dissected from the ligule. This tissue includes a meristem with dividing and elongating cells. The tissue was kept at − 80 °C for each sample. Total RNA of 100 mg of 24 leaf tissue sample pools (2 genotypes × 2 treatments × 2 times of sampling × 3 replicates) was isolated using TRIzol (Invitrogen, USA) and purified using the Qiagen RNeasy MinElute Cleanup Kit column according to the manufacturer’s instructions.

The RNA was sequenced using Illumina HiSeq 2500 at the Mount Sinai Hospital in Toronto, Canada, obtaining 8.4 to 12 million 100-nucleotide paired reads per sample (Additional file1: Table S1). FastQC v0.11.3 was used to assess the quality of the reads and identify the presence of adapters. TopHat v2.1.0 released 6/29/2015 [38] was used to align the RNA-Seq reads to the maize reference B73 genome version AGPv3.

### Tests of Tophat alignment parameters and final alignment criteria

RNA-Seq reads should align to the reference genome locus that is the same as the locus that gives rise to the read. However, if the criteria to align a cDNA sequence to the reference genome are strict, allelic differences between genes may prevent reads from aligning. As read alignment criteria are loosened, more reads correctly align to their reference genome positions. However, the number of reads aligning to incorrect loci is also expected to increase.

We evaluated 17 different sets of TopHat parameters to explore criteria that balanced capturing reads from the same genes with capturing reads from different genes. We varied parameters that limit the edit distance and the total numbers of allowed mismatches and gaps in the final read alignments (Additional file 2: Table S2). TopHat uses Bowtie2 to seed alignments with read subsequences prior to extension. We did not alter the criteria that are used to seed alignments Alignment tests were first performed with the RNA-Seq reads of the CG60_D1_Stress_R3 sample (sample 1) and then included CG102 and control growth conditions.

For each alignment criteria, we recorded the percentage of all reads that concordantly aligned, e.g. both reads of a paired-end read mapped to the same genomic region. We also counted read pairs with multiple, equally good alignment positions. Finally, we counted discordant reads that have mate-pairs that align in different genomic regions. Focusing on reads that mapped to maize chromosome 1, we used Samtools (v1.2) and bcftools (v1.2) [39] to identify polymorphisms between aligned reads and the maize B73 reference. We also identified nucleotide differences among reads that mapped to a single genomic position. Reads that map to a common reference genome site but have nucleotide differences may be from paralogous sites or loci that are heterozygous. They may also be due to sequencing errors. CG60 and CG102 are inbred lines and thus highly homozygous.

### Estimating transcript abundance and identifying differentially expressed genes

Reads from all 24 samples were aligned to the reference maize genome AGPv3 as annotated in Zea_mays.AGPv3.22.gtf. Based on the tests described above, we used the --read-mismatches 20, −-read-gap-length 20, −-read-edit-dist 20 arguments for TopHat alignment. One sample (CG60_D1_Control_R1, sample 18) was discarded from further analyses due to an unusually low percentage of paired-end reads with concordant mapping alignments (69.2%), and an unusually high percentage of read pairs with multiple alignments (51.1%) and discordant mapping (27.8%) (Additional file 1: Table S1, Additional file 2: Table S2). Using the TopHat derived .bam file, HTSeq (v0.6.1) [40] estimated the number of reads aligning to gene models based on Zea_mays.AGPv3.27.gff3. CG60 and CG102 reads mapped to a similar number of gene models. For example, among the three CG60 and CG102 D4 control replicates, an average of 27,222 and 27,026 gene models have at least one mapped read, respectively. We removed genes from the data set when fewer than three samples (out of the 11 samples for D1 and 12 samples for D4) had at least one count per million mapped reads using the EdgeR cpm function. Out of the 39,469 annotated genes, 22,942 and 22,867 were kept for time point D1 and time point D4, respectively. The remaining read counts for both time points were normalized using the calcNormFactors function of EdgeR. This function uses the “trimmed mean of M-values” normalization method [41].

We used EdgeR [42] to determine if temperature, genotype, and specific temperature and genotype combinations significantly explain transcript abundance variation. We analyzed D1 and D4 samples separately and used the following model:

$$ {\mathrm{Y}}_{\mathrm{i}\mathrm{j}\mathrm{k}}=\mathrm{u}+{\mathrm{G}}_{\mathrm{i}}+{\mathrm{T}}_{\mathrm{j}}+{\mathrm{R}}_{\mathrm{k}}+{\left({\mathrm{G}}^{\ast}\mathrm{T}\right)}_{\mathrm{i}\mathrm{j}}+{\mathrm{e}}_{\mathrm{i}\mathrm{j}\mathrm{k}} $$

Y_ijk_ is the transcript abundance for the genotype i, that received treatment j (cold or control temperature growing conditions) in the kth replication. G_i_ is the effect of the genotype i (CG60 or CG102), T_j_ is the effect of the treatment j (cold stress or control), R_k_ is the effect of the replicate k in time, and (G*T)_ij_ is the effect of the interaction of genotype i with treatment j, e_ijk_ is the random error.

The EdgeR function estimateGLMCommonDisp was used to estimate a common dispersion for the genes, and a false discovery rate (FDR) correction of 0.05 was used to generate lists of genes with significant effects. We tested FDR values of 10%, 5 1% and 0.1% for the differentially expressed genes. Results from these analyses are in supplemental information. Differentially expressed genes include genes that have reads mapped from only one inbred line. These genes may represent presence/ absence variants. EdgeR’s plotMDS function was used to generate multi-dimensional scaling plots of the RNA samples. The method used the 500 genes with the largest log2 fold-differences in read counts between each pair of samples to determine sample distance.

### Inferring the functions of differentially expressed genes

We used the agriGO analysis tool [43] to identify those gene ontology (GO) terms significantly overrepresented among differentially expressed genes using Fisher’s Exact Test and the Benjamini-Yekutieli procedure to control the false discovery rate at 0.05 [44]. Of the 22,942 and 22,867 genes expressed in our D1 and D4 samples after filtering, 16,265 and 16,134 have GO annotations. The frequencies of GO terms amongst these expressed genes represent their expected frequencies among differentially expressed genes. To identify gene models that are transcription factors, we used GrassTFDB [45]. To associate gene models with metabolic pathways, we used MaizeCyc2.2, available from gramene [46]. As with GO annotations, Fisher’s Exact Test was used to identify pathways over or underrepresented in subsets of expressed genes relative to all expressed genes. The results were adjusted for multiple hypotheses testing using the Benjamini-Hochberg correction with a 5% FDR.

## Results and discussion

### Inbred lines stably affect the transcriptome, and cold has a transitory effect

**Fig. 2:**
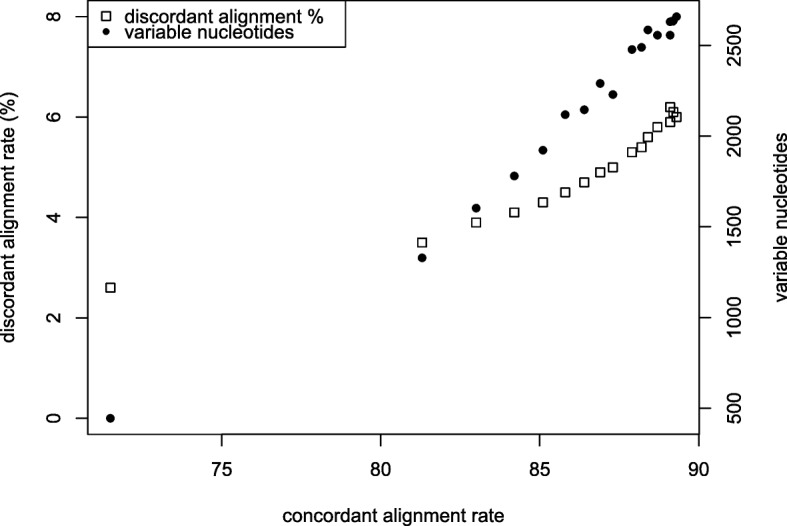
The effects of alignment parameters on alignment attributes. As the frequency of concordant read alignments increases (x-axis), the frequency of discordant paired end read alignments increases (left y axis) and the number of variable nucleotides at single genomic positions increases (right y-axis). Alignment criteria set nine, the criteria set used in this study, results in concordant frequency of 87.3%, discordant frequency of 5%, and 2229 variable nucleotides. The data is from sample 1 chromosome 1 alignments

This study investigates the transcriptional response of two inbred lines to cold temperatures and to the alleviation of cold temperatures (Fig. 1). To evaluate the contributions of cold stress on the inbreds’ transcriptomes, we aligned the RNA-Seq reads from the inbred lines to the B73 maize reference genome. We expected some RNA-Seq reads from the inbred lines would not align to the reference genome because of sequence divergence between genes, thereby underestimating transcript abundance. Simulations have illustrated that nucleotide and indel polymorphisms can prevent reads from mapping [47]. Maize also exhibits one of the highest nucleotide diversities among crop plants, an average of 1% divergence between sequences, along with high levels of copy number and presence/absence variation [48–51]. We thus investigated the effects of different alignment criteria on chromosome one read pair mapping. We viewed concordant alignments, where both reads of paired-end sequences align to nearby regions of the reference genome, as likely reflecting true positive alignments. Moderately reducing the alignment stringency greatly increases the number of concordantly aligned reads. With sequencing data from one inbred growing in warm temperatures, changing the number of allowed mismatches, edit distance, and gaps per alignment to six from the default value of two, causes a 9.8% increase in concordant alignments from 71.5 to 81.3% (Additional file 2: Table S2). The proportion of concordant alignments slowly increases and tapers off with further reductions in alignment stringency. Reducing the stringency of alignment may increase the alignment frequency of paralogous reads [48]. We viewed both nucleotide differences amongst reads aligned to the same genomic position and discordant alignments as indicators of paralogous read alignment. The proportion of discordantly aligned reads increases from 2.6 to 3.5%, when changing an edit distance from two to six, rises close to linearly (slope circa 0.3%) with increases in the concordant alignment rate (Additional file 2: Table S2, Fig. 2). The number of nucleotide sites that differ among aligned reads to the same genomic position increases from 445 to 1329 with the moderate reduction in stringency, and these sites also increase linearly with increases in convergent mapped pairs. Changing alignment parameters has similar effects on reads from different inbred lines grown in different conditions (Additional file 3: Table S3, Additional file 4: Figure S1). To balance capturing new, true positive alignments with false positive alignments, we chose an intermediate criteria set as the maximum alignment differences allowed for reads (Additional file 2: Table S2, Fig. 2).

**Fig. 3:**
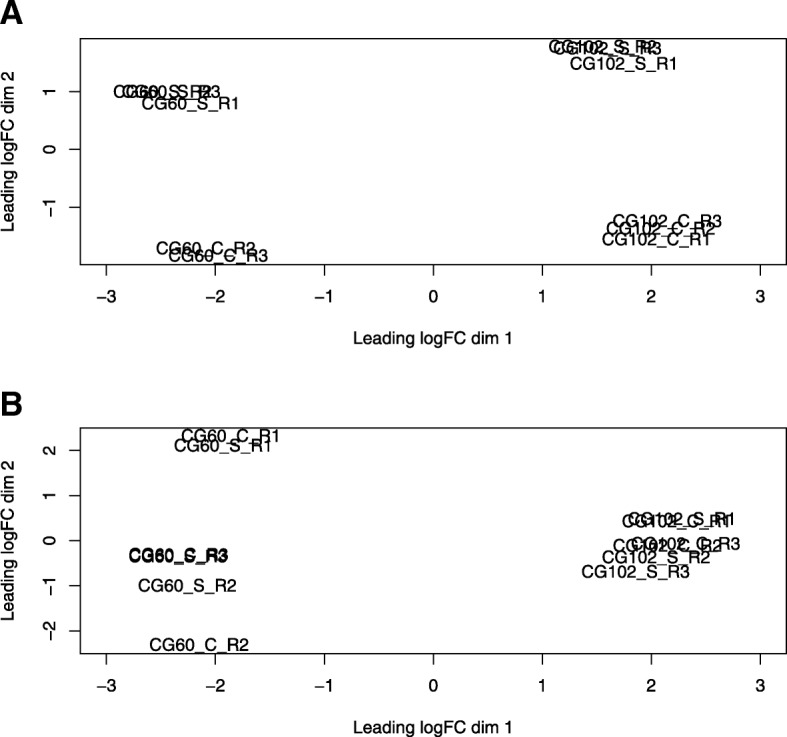
Multidimensional scaling (MDS) representations of distances between gene transcript abundance estimates. **a** Projections of samples from plants grown at low temperatures (14 °C/2 °C) for 24 h, and their controls grown continuously at 24 °C/14 °C. **b** Projections of samples from plants grown for 24 h at 24 °C/14 °C following a 72 h exposure to 14 °C/2 °C stress. Plants’ controls were grown continuously at 24 °C/14 °C. Samples are labelled as CG60/CG102_stressed/control plants_R1/R2/R3, where R is replicate

We used multi-dimensional scaling to investigate the global similarities in gene expression among CG60 and CG102 plants grown in cold for 24 h (14 °C day / 2 °C night) and exposed to warmer temperatures (24 °C / 14 °C) for 24 h after cold exposure (24 °C / 14 °C) (Fig. 1). Experiments were replicated over three separate time periods. In Fig. 3a and b, the transcript abundance differences of each sample pair’s 500 most divergent genes are represented as a distance between samples on two dimensions [52, 53]. In cold grown plants and their controls, primarily genotype and secondarily temperature explain sample transcript abundance differences (Fig. 3a). Genotype is the major factor, as the distance between CG60 and CG102 samples on the horizontal axis is about twice the distance between cold exposed and control samples on the vertical axis. Replicates of the same genotype and temperature treatment cluster together. Following 72 h cold treatment and 24 h recovery, genotype is again a major factor distinguishing samples (Fig. 3b). However, the strong transcriptome response to low temperatures is transitory. Past exposure to cold is unrelated to sample distance in the MDS plot, and the third axis also does not separate samples based on temperature treatment (data not shown). After genotype, replicate best accounts for transcript abundance variation. The CG60 transcriptome varies more across replicates than does the CG102 transcriptome (Fig. 3b).

A large number of transcripts have significant genotype main effects in cold grown and recovering plants. We fit a linear model to estimate the effects of genotype, treatment, and their interactions on each gene’s transcript abundance. The model was separately applied to data from plants grown in cold and their controls (D1) and plants with past cold exposure and their controls (D4, Fig. 1). In cold, 6514 genes (29% of 22,942 expressed genes) have significantly different transcript abundances between CG60 and CG102 (FDR *P* < 0.05). After cold exposure, 7694 genes (34% of 22,867 expressed genes,) differ significantly between genotypes (Table 1). With more stringent FDR, the number of differentially expressed genes is smaller but still large (Additional file 5: Table S4). In both stress and recovery conditions, 4647 genes are differentially expressed between genotypes (FDR P < 0.05), representing 71% and 60% of the genes with significant genotype effects in days 1 and 4, respectively. For 99% of these 4647 genes, the same genotype has the higher transcript abundances in both days. Thus, most genotypic effects on expression levels are similar regardless of temperature. Studies of a broad range of genotypes across multiple growing conditions could address if consistent genetic effects on gene expression are the norm. Some work suggests our level of genotypic stability is unusual. For example, in wheat, *Triticum aestivum*, for 1950 genes the effect of one genotype on a gene’s transcript abundance differed from a second genotype in at least one of two light regimens. Genotype differences were consistent for only 379 (19%) genes across light regimens [54].

**Table 1:** Numbers of differentially expressed genes

Time	Total genes	Genes after filtering	Treatment	Genotype	Interaction
D1	39,469	22,942	10,549 (695 TF^a^)	6514 (309 TF)	1541 (124 TF^a^)
D4	39,469	22,867	556 (29 TF)	7694 (379 TF)	323 (11 TF)

^a^Significantly enriched for transcription factors (TF) (Fisher Exact Test, FDR *p* value< 0.05) relative to the background (1233 TF in the 22,942 expressed genes in D1 and 1201 TF in the 22,867 expressed genes in D4)

Temperature affects the transcript abundances of a notably higher number of genes than does genotype. Transcript abundances significantly differ for 10,549 of 22,942 expressed genes (46%) in cold-grown plants relative to control plants (Table 1). This number of genes is higher than reported in previous studies. For example, in two studies 17% and 30% of microarray probes differed in response to cold [16, 21]. We suggest the high number of genes with significant temperature responses is a function of the inbred lines, as well as the high sensitivity of RNA-Seq technology. Recently, Waters et al. (2017) found that temperature explained expression variation for 41% of genes in a comparison of inbreds B73 and OH43. Here, genotype explains most expression variation in the multidimensional scaling plot (Fig. 3), yet there is a larger number of genes with treatment effects than genotype effects (Table 1). The transcriptome’s response to environmental differences is broad and shallow relative to its response to genotypic differences.

The transcriptomes of recovering plants are similar to the transcriptomes of plants unexposed to cold. In plants that had been exposed to cold relative to plants grown in control conditions, only 556 of 22,867 expressed genes (2.4%) have significantly different treatment effects (Table 1). These results are consistent with the idea that plant transcriptomes recover quickly after exposure to cold temperatures. For example, Zhang et al. [22] applied a more severe stress to rice seedlings, and compared transcriptomes of rice seedlings continuously exposed to 29 °C with seedlings exposed to 4 °C for 2 days followed by 1 day at 29 °C. Two rice cultivars assayed with a 51,279 gene microarray, had 445 genes and 3007 genes differentially expressed between recovery and control plants.

### Cold temperatures increase transcript abundances of many transcriptional regulators, signalling molecules, cytokinin glucoside biosynthesis enzymes, and proteins that respond to environmental stimuli

**Fig. 4:**
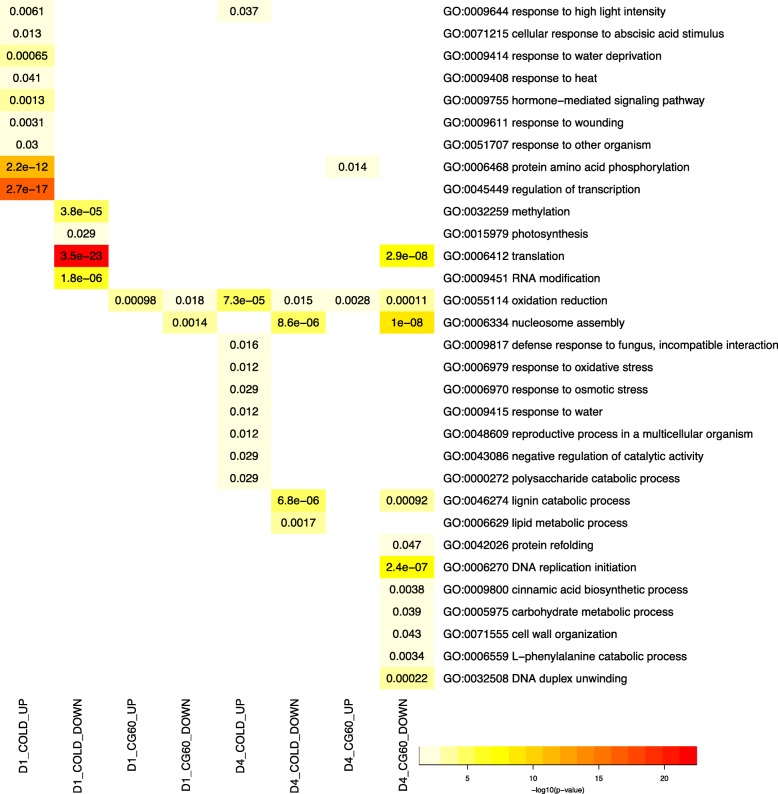
Biological process GO terms over-represented (Fisher Test, Benjamini-Hochberg adjusted *p*-value < 0.05) in upregulated (UP) and downregulated (DOWN) genes. D1 COLD-UP and COLD-DOWN refer to genes significantly up or down regulated in plants exposed to 24 h of cold, relative to controls; D4 COLD-UP and COLD-DOWN refer to genes significantly up or down regulated in plants exposed to 72 h of cold relative and 24 h of recovery, relative to controls. CG60-UP refers to transcripts high in inbred CG60 relative to CG102. The *P* values of significantly enriched GO terms with no significant child terms are shown

Cold treated plants had significantly higher transcript abundances for 5459 genes. Notably, cytokinin-O-glucoside biosynthesis pathway genes are enriched amongst genes up-regulated in cold (Table 2). Cytokinins are key regulators of abiotic stress responses [55–57], and they control processes including photosynthesis [58], leaf senescence [59], and cell division [56]. Glucosylation permanently deactivates cytokinin or generates an inactive storage form [56]. Wheat plants grown at a constant 5 °C had over eight-fold higher levels of inactive cytokinin glucoconjugates than do wheat plants grown in 20 °C /18 °C [54]. Of 72 cytokinin-O-glucoside biosynthesis pathway genes expressed in our samples, 37 are upregulated. Among these upregulated genes, seven putative cytokinin-O-glucosyltransferases have positive cold effects. These genes are cytokinin-O-glucosyltransferases 1 (GRMZM2G056335, GRMZM2G007012), 2 (GRMZM2G041699 GRMZM2G083130, GRMZM2G373124), and 3 (GRMZM2G338465, GRMZM5G854655). Cold-induction of these genes’ transcripts very likely contributes to cold-induced cytokinin glucoconjugate accumulation. We investigated these genes’ attributes. Most strikingly, many genes with higher transcript levels in cold grown plants than control grown plants encode transcription factors, kinases, and phosphatases (Fig. 4, Additional file 6: Figure S2, Additional file 7: Table S5). The enrichment of transcription factors amongst upregulated transcripts has been widely reported (e.g. [16, 29]), yet the number of up-regulated transcription factors identified here is remarkably high. Of the 458 genes expressed in cold and control grown plants and annotated with the GO biological process term “transcription factor activity,” close to one-half (212) have significantly higher transcript abundances due to cold. For example, DRE binding factor 1 (DBF1/DREB1; GRMZM2G061487) and DREB2B (GRMZM2G006745) have been previously identified as cold induced [60, 61]. Transcripts of these transcription factors and two additional putative DRE transcription factors, DREB1 (GRMZM5G889719) and DREB4 (GRMZM2G380377), increase in the cold treated plants. Cold significantly enhances the abundances of 38% of expressed genes with protein serine/threonine kinase annotations (289 of 759), and 50% of expressed genes with serine/threonine phosphatase annotations (28 of 56). For example, cold has a positive effect on the transcript abundances (*P* < 1.0 e-08) of the three maize genes with high sequence similarity to the rice calcium dependent protein kinase7 (GRMZM2G321239, GRMZM2G314396, and GRMZM2G81310). OsCDPK-7 is upregulated in cold temperatures [62]. Suppressing of endogenous transcripts in rice led to an increased cold sensitivity [63], and ectopic expression of *Os*CDPK-7 in transgenic rice (though not in sorghum) enhanced cold tolerance [63, 64].

**Table 2:** Overrepresented pathways in upregulated (UP) and downregulated (DOWN) genes across growth conditions and genotypes

Pattern^a^	Pathway id	Pathway name	In group	In all genes	Bh_p_val
D1_COLD_UP	PWY-2902	cytokinins-*O*-glucoside biosynthesis	37	72	0.0002
	PWY-3781	aerobic respiration -- electron donor II^a^	7	106	0.0012
D1_CG60_DOWN	PWY-695	abscisic acid biosynthesis	9	12	0.0013
	PWY-6299	aldehyde oxidation I	6	8	0.0354
	PWY-6446	benzoate biosynthesis III (CoA-dependent, non- β -oxidative)	8	15	0.0354
	PWY-6444	benzoate biosynthesis II (CoA-independent, non- β -oxidative)	8	15	0.0354
	PWYQT-4429	CO_2_ fixation into oxaloacetate	4	4	0.0354
D4_COLD_DOWN	PWY-282	cuticular wax biosynthesis^b^	0	5	0.0057
D4_COLD_UP	PWY-282	cuticular wax biosynthesis	3	5	0.0115
D4_CG60_DOWN	PWY-6446	benzoate biosynthesis III (CoA-dependent, non- β -oxidative)	10	15	0.0091
	PWY-6444	benzoate biosynthesis II (CoA-independent, non- β -oxidative)	10	15	0.0091
	PWY-724	superpathway of lysine, threonine and methionine biosynthesis II	23	59	0.0168
	PWY-6457	*trans*-cinnamoyl-CoA biosynthesis	6	7	0.0220
	PWY-5156	superpathway of fatty acid biosynthesis II (plant)	18	44	0.0332
	PWY-1081	homogalacturonan degradation	18	45	0.0332
	TRPSYN-PWY	tryptophan biosynthesis	11	22	0.0332
	PWY1F-467	phenylpropanoid biosynthesis, initial reactions	6	8	0.0332
	PWY-5172	acetyl-CoA biosynthesis (from citrate)	6	8	0.0332
	PWY0–162	pyrimidine ribonucleotides de novo biosynthesis	14	33	0.0391
	PWY-5670	epoxysqualene biosynthesis	4	4	0.0429

^a^The code for the gene expression pattern is “day”, main effect factor level, direction of the main effect. D1 refers to samples harvested during cold exposure and their controls. D4 refers to samples harvested 24 h after cold exposure and their controls. CG60_UP indicates a significant, positive effect of CG60. CG60_DOWN indicates a significant, negative effect of CG60

^b^Pathways with b at the end of the description were underrepresented amongst the gene set

Cold also induces stress response components that are shared across both abiotic and biotic stress response pathways. Up-regulated genes in cold are enriched with GO terms “response to high light intensity” (28 genes), “response to water deprivation” (85 genes), “response to heat” (55 genes), “response to wounding” (55 genes) and “response to other organisms” (167 genes) (Fig. 4, Additional file 7: Table S5). Plants exposed to drought, herbivory, and infection share some common transcriptome responses [23, 65]. The commonalities of transcriptome responses among stresses may in part be due to shared countermeasures against increased reactive oxygen species (ROS) levels [66]. Scavengers of ROS include ascorbate and glutathione. Ascorbate peroxidase oxidizes ascorbate to the monodehydroascorbate radical (MDHA) in the presence of H_2_O_2_, and monodehydroascorbate reductase reduces MDHA back to ascorbate. Relative to control, cold increases transcript levels of one of three putative ascorbate peroxidases and all of the three putative monodehydroascorbate reductases (GRMZM2G093346, GRMZM2G084881, GRMZM2G087259, and GRMZM2G134708, respectively; Additional file 7: Table S5). Cold temperatures enhance transcript levels of a putative glutathione synthetase (GRMZM2G155974) and a putative gamma-glutamylcysteine synthetase (GRMZM2G111579) (Additional file 7: Table S5). The latter is a rate limiting step in glutathione biosynthesis [67–69].

### Transcript abundances of many genes involved in photosynthesis, cellular replication, and protein biosynthesis are downregulated in cold-grown plants

**Fig. 5:**
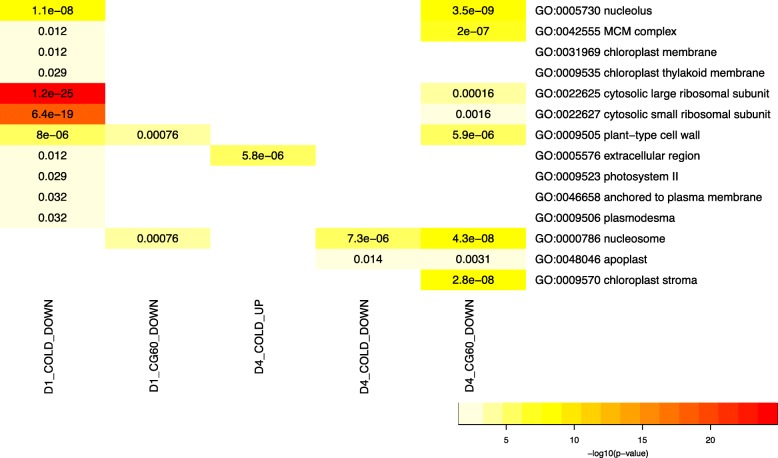
Cellular component GO terms over-represented (Fisher Test, Benjamini-Hochberg adjusted p-value < 0.05) in upregulated (UP) and downregulated (DOWN) genes. D1 COLD-UP and COLD-DOWN refer to genes significantly up or down regulated in cold-grown plants relative to controls; D4 COLD-UP and COLD-DOWN refer to genes significantly up or down regulated in plants that had been exposed to cold relative to controls. CG60-UP refers to transcripts high in inbred CG60 relative to CG102. The P values of significantly enriched GO terms with no significant child terms are shown

Of the 10,549 genes with transcripts affected by cold, 5090 have reduced transcript levels. Protein biosynthesis, and DNA replication associated transcripts are highly represented amongst them. Highly enriched translation-associated GO terms include “translation”, “cytosolic small ribosomal subunit”, “nucleolus”, and “cytosolic large ribosomal subunit” (Figs. 4 and 5; Additional file 8: Table S6). The MCM complex primes chromatin for DNA replication, and transcripts of its composite proteins are highly expressed in dividing cells [70]. Eight out of the 11 expressed genes annotated with the “MCM complex” cellular component GO term are down regulated in the cold treated samples (*P* < 0.001; Fig. 5; Additional file 8: Table S6).

Cold induced reduction of photosynthesis-related transcripts has been widely reported, correlating both with lower chlorophyll content and photosynthetic enzyme activity [16, 20, 71]. We find genes associated with photosynthesis, chloroplasts, and photosynthesis-specialized cells are preferentially down-regulated in cold. Genes with photosynthesis related GO terms are over-represented amongst the genes down-regulated under cold stress (Fig. 4; Additional file 8: Table S6). Gene products localized to the chloroplast are preferentially downregulated (Fig. 5; Additional file 8: Table S6). The cellular component GO terms: “chloroplast membrane”, “chloroplast thylakoid membrane” and “chloroplast stroma” are all overrepresented among genes down regulated by cold (Fig. 5; Additional file 8: Table S6). Out of 15 expressed genes from the chloroplast genome (Pt), seven are downregulated in cold treated samples. Finally, downregulated genes are also enriched for localization to bundle sheath and mesophyll cells. In maize, the primary carboxylation of photosynthesis is catalyzed by phosphoenolpyruvate carboxylase in mesophyll cells, and the captured CO_2_ is used within the Calvin cycle localized to the bundle sheath cells. Recent work has identified transcripts preferentially expressed in bundle sheath (BS) cells relative to mesophyll (M) cells, and vice versa [72]. As transcripts that differ in abundance between BS cell and M cells likely play roles in photosynthesis, we anticipated both sets would be down-regulated in cold temperatures. Indeed, cold affects the 801 genes with high transcript levels in BS cells and the 792 genes with high transcript levels in the M cells at a higher frequency than cold affects other transcripts. Twenty-two percent of transcripts not preferentially expressed in BS and or M cells are down regulated by cold. In contrast, 31% percent of transcripts preferentially expressed in either BS or M are down-regulated (*P* < 0.0001; Additional file 7: Table S5; Additional file 9: Figure S3).

### Genotype-specific responses to cold have a small global effect on the transcriptome yet affect transcripts of discrete cellular processes

Genotype x temperature interactions contribute notably less to transcriptome variation than do genotype and temperature main effects (Fig. 3a; Table 1). In the MDS plot (Fig. 3a), genotype and treatment correspond to the two primary axes, and y-axis distances between the cold stress and control samples and x-axis distances between CG60 and CG102 are similar. Despite its relatively small effect on global expression variation, the genotype x temperature model term is significant for 1541 genes (FDR *P* < 0.05; Table 1). The numbers of genes whose transcripts differ due to genotype and temperature are consistently larger than the number of genes that differ due to genotype x temperature [16, 29].

Genes whose genotypic transcript abundance differences are conditioned on temperature are enriched for a number of GO molecular function and biological process terms. The enriched processes overlap little with previous studies. Enriched biological process GO terms include oxidation reduction (GO:00055114), response to hydrogen peroxide (GO:00042542), and response to heat (GO:0009408) (Additional file 10: Table S7). GO molecular functions include transcription factor regulator activity (GO:0030528), oxidoreductase activity (GO:0016491), serine-type endopeptidase inhibitor activity (GO:000486), and iron ion binding (GO:0005506) (Additional file 10: Table S7). Transcription factors with significant genotype by temperature interactions include nine AP2/EREBP, 11 NAC, 13 MYB, one Tify, and four WRKY family members (Additional file 7: Table S5). Two AP2/EREBP genes encode DREBs (GRMZM2G006745, GRMZM2G061487). In cold- exposed rice, enriched processes among genes with genotype x environment effects include photosynthesis and auxin responsiveness, and enriched functions include leucine rich repeat proteins, transcription factors, and thioredoxin family proteins [22]. Members of the same transcription factor families have rice genotype-specific responses to chilling temperatures [22]. In maize, enriched processes include photosynthesis, signal transduction, and carbohydrate and amino acid metabolism, among others. Sobkowiak et al. [16] reported 66 genes with genotype-specific responses to cold. Not one gene of the 58 with GRMZM codes is among the 1541 genes identified in this study. The observation that genes with significant genotype x temperature interactions overlap little if at all between studies indicates cold temperature responses are due to multiple regulatory factors.

**Fig. 6:**
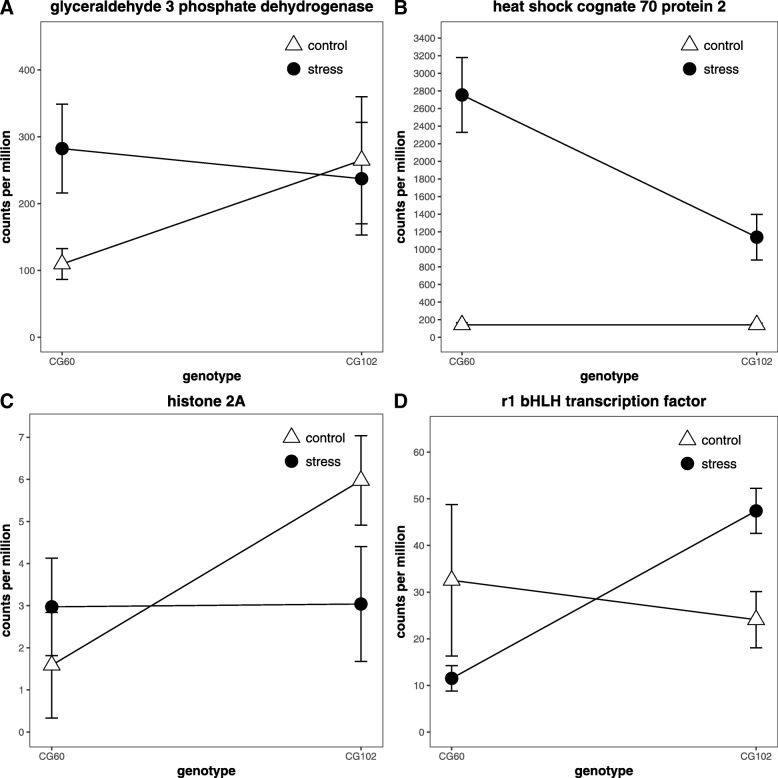
Examples of gene transcripts whose responses to cold differ between inbred lines. **a** and **b** plot the transcript abundances of two genes from control plants and plants grown 24 h in cold temperatures. **c** and **d** plot the transcript abundances of two genes from control plants and plants grown 24 h after the end of cold temperature exposure. A putative glyceraldehyde-3-phosphate dehydrogenase, GRMZM2G071630, (**a**) and a putative heat shock cognate 70 kDa protein 2, GRMZM2G428391, (**b**) increase in cold grown CG60 relative to control CG60 but do not increase in cold-grown CG102. These two genes are representative of the transcript changes amongst genes responsive to hydrogen peroxide (GO:00424542). (**c**) A histone H2A gene (GRMZM2G056231) is greatly downregulated in CG102 plants with past cold exposure relative to control plants, but it is not downregulated in CG60 plants with past cold exposure. This gene’s pattern is typical of genes involved in DNA replication and cell division. (**d**) The transcription factor *R1* (GRMZM5G822829) is up-regulated in CG102 with past cold exposure but not up-regulated in CG60 with past cold exposure. A similar pattern was observed with other anthocyanin biosynthesis genes. Error bars represent the standard error

Genes with genotype-specific responses to environmental changes are sometimes considered to be candidate genes that explain phenotypic variation [16, 22]. However, environmental changes are known to uncover mostly neutral or maladaptive genetic variation that has not been exposed to selection [32]. The over-representation of functionally similar genes may be because genetic variation in these genes is less subject to purifying selection than genetic variation in other genes. To address this possibility, we investigated the GO group GO:00424542, “response to hydrogen peroxide” to determine if genetically variable genes involved in the same biological process are consistently up-regulated in a single line in response to cold. Of 12 hydrogen peroxide response genes whose transcripts increase in cold, the abundances of nine genes increase in CG60 and do not increase or increase less in CG102 (Additional file 11: Table S8). Most genes have a large difference in cold response. For example, two putative glyceraldehyde-3-phosphate dehydrogenases (GAPDH; EC 1.2.1.12; GRMZM2G176307, GRMZM2G071630) have much higher transcript levels in cold grown CG60 relative to its control and have lower transcript levels in cold grown CG102 relative to its control (Fig. 6a; Additional file 11: Table S8). Steady-state GAPDH mRNA levels increased in cold-treated wheat plants [73], and our maize genes are most similar to the *Arabidopsis thaliana* cytosolic GAPDH that transduces signals from and suppresses reactive oxygen species [74, 75]. Three putative heat shock protein encoding genes, one HSP20 (GRMZM2G034157) and two HSP70s (GRMZM2G024718, GRMZM2G428391) are also upregulated more in CG60 in response to cold than in CG102 (Additional file 11: Table S8; Fig. 6b). Heat shock proteins play a central role in protecting proteins from temperature induced denaturation and aggregation. Finally, myoinositol has been proposed to protect plants from abiotic stresses [76]. A putative myoinositol-1-phosphate synthase (GRMZM2G155242) transcript is more abundant in cold-grown CG60 than in CG102 (Additional file 11: Table S8). It is unlikely that many CG60 hydrogen peroxide response genes would be upregulated relative to CG102 genes by chance, and this pattern of genotype-specific environmental responses suggests functional significance. An eQTL mapping study could elucidate if a single, large effect trans regulatory eQTL, and/or many cis-acting eQTL [77] cause the consistent cold-induced up-regulation of hydrogen peroxide response transcripts in CG60 relative to CG102.

### Transcripts up-regulated both during and after cold exposure have different functions than transcripts up-regulated only after cold exposure

The recovery transcriptome has global similarity to the control transcriptome across inbred lines (Fig. 3b). Nonetheless, transcripts of 310 genes are more abundant in plants 24 h after cold exposure than in control plants (56% of the 556 differentially expressed genes, Table 1). These 310 genes are enriched for a number of GO categories (Fig. 4; Additional file 8: Table S6) and a single pathway, “cuticular wax biosynthesis.” We sub-divided up-regulated genes into those genes that are also up-regulated during cold temperature growth and those genes that are only up-regulated in the recovery period.

160 up-regulated genes (52%) are upregulated in cold grown plants (D1), indicating these transcripts do not reset to normal levels after cold temperature exposure. These 160 genes are enriched for 17 GO biological process terms (Additional file 12: Table S9). Fifteen terms involve environmental responses including “response to cold,” “response to high light intensity,” “response to water,” and “response to osmotic stress.” A number of ABA and H_2_O_2_ responsive genes are amongst the shared genes. These include RARE COLD INDUCUBLE 2A (RCI2A; GRMZM2G015605), HIGHLY ABA-INDUCED PP2C GENE 2, HAI2; GRMZM2G059453), and a glutathione S-transferase encoding gene (Tau 25A; GRMZM2G308687). In Arabidopsis, RCI2A transcript levels increase when plants are exposed to cold temperatures, and the gene is co-regulated with abscisic acid responsive genes [78]. Tau 25A has very high up-regulation in plants induced to produce chloroplastic H_2_O_2_ [79]. Other genes upregulated in both cold and recovery conditions include NaCl stress protein1 (GRMZM2G015605), glutathione transferase5 (GRMZM2G308687), and monodehydroascorbate reductase (GRMZM2G134708). In contrast to environmental response genes, transcripts of signaling and regulatory network molecules rapidly reset to control levels. GO terms “protein amino acid phosphorylation” and “regulation of transcription” are significantly enriched amongst genes upregulated during cold (Fig. 4; P < E-12) but are not significantly enriched amongst up-regulated genes shared between cold and recovery plants (Additional file 12: Table S9).

One-hundred fifty genes (48%) are upregulated only in recovering plants. This de novo transcriptional regulation increases transcripts of genes for protease control, lipid degradation, and cuticular wax biosynthesis (Additional file 12: Table S9). Five of the eight enriched molecular function terms are related to enzymatic activity regulation (Additional file 12: Table S9). Six up-regulated genes have serine-type endopeptidase inhibitor activity annotations. The de novo up-regulation of protease inhibitors following cold exposure indicates recovering plants actively curtail protease activity. Proteases may have been activated in cold temperatures due to the increase of reactive oxygen species and the decrease in active cytokinin, and the up-regulation of protease control genes in the recovery period signals a return to a normal state. Among the 11 genes annotated with the “lipid metabolic process” GO term, six genes encode putative lipases that break lipids down to fatty acids. Lipid derived polymers accumulate within grass leaves exposed to low temperatures [80]. Our result suggests these lipids are broken down in recovering plants. Finally, cuticular wax can both act as a photoprotective layer and prevent non stomatal water loss, and its synthesis increases in response to water stress in maize [81, 82]. A putative octadecanal decarbonylase (GRMZM2G029912) cuticular wax biosynthesis gene is up-regulated only in the recovery period. Increasing the transcript levels of the rice GMRZM2G029912 homolog increased cuticular wax accumulation and decreased water loss [83]. A second up-regulated cuticular wax biosynthesis gene, a putative diacylglycerol O-acyltransferase (GRMZM2G077375), is also only up-regulated in the recovery period. Other genes including a homolog of MYB94 (GRMZM2G055158) that activates Arabidopsis, cuticular wax biosynthesis and another octadecanal decarbonylase GRMZM2G083526 are up-regulated in both recovery and control conditions (Additional file 7: Table S5) [84].

### Transcripts down-regulated both during and after cold exposure have different functions than transcripts down-regulated only after cold exposure

For 246 genes (44% of the 556 differentially expressed genes in plants following cold treatment, Table 1), transcripts in recovering leaves are lower than in control leaves. A number of GO categories are over-represented amongst the down-regulated genes (Fig. 4). Close to one-half of the 246 genes, 120 genes, also have low transcript abundances within cold-grown plants. Transcripts associated with cell division, chromatin, and translation remain repressed in recovering plants (Additional file 12: Table S9). For example, five histone encoding genes, classified within the nucleosome assembly GO category (GO:0071103), are down-regulated in both cold temperature-grown and recovering plants. These genes are histone H2B.2 (GRMZM2G119071), histone H2B.5 (GRMZM2G342515), histone H3.2 (GRMZM2G179005) and histone H4 (GRMZM2G084195 and GRMZM2G479684) (Additional file 7: Table S5). As histone transcript abundance levels are tightly correlated with cell cycle and cell proliferation [85, 86], these results suggest recovering plants have slower cell division than control plants. Interestingly, the 120 genes downregulated both during and after cold are not enriched for photosynthesis related genes. This result suggests that cold’s negative effect on photosynthesis genes’ transcripts is transitory.

51% of all down-regulated genes (126 genes) are down-regulated only in plants recovering from past exposure to cold. These genes are enriched for 33 biological process terms, six molecular function terms, and six cellular component terms (Additional file 13: Table S10). We sample leaves 24 h after cold and expect that recovery-specific transcripts will become fewer and fewer over time as control and treated plants become more similar. Notably, lignin and suberin biosynthesis transcripts are preferentially down-regulated only after recovery from cold exposure begins (Additional file 13: Table S10). The oxidative polymerization of monolignols into lignins involves laccases, *p*-diphenol: dioxygen oxidoreductases (EC 1.10.3.2). Five putative laccases within the “lignin catabolic process” GO term are down-regulated in recovering plants. Four of the five downregulated laccases are most similar to Arabidopsis LAC2 and LAC17. Both genes contribute to lignin biosynthesis in Arabidopsis [87, 88], and their transcript reduction enhances cold tolerance [89, 90]. Although miR397 targets both LAC2 and LAC17 transcripts in Arabidopsis, the miRNA sequence is not conserved across the four maize homologs. Downregulation of suberin biosynthesis genes is likely because, as noted above, exposure to cold induces accumulation of lipid derived polymers. These polymers are comprised in large part of cutin/suberin like 18-hydroxy-9,10-epoxyoctadecanoic acid [80]. Downregulated suberin biosynthesis transcripts include four well-characterized genes. A putative feruloyl-CoA transferase (AC155610.2_FG007) is required for suberin synthesis in Arabidopsis (AT5G41040). A putative glycerol-3-phosphate acyl transferase (GRMZM2G166176) encodes a step in suberin biosynthesis in which C16 or C18 fatty acids are elongated up to C30 fatty acids (LOC_Os05g38350). A cytochrome P450 (GRMZM2G020500) is associated with saturated fatty acid elongation and suberin production (LOC_Os04g47250) [91]. Finally, disruption of the Arabidopsis homolog of a putative acyl-acyl carrier protein thioesterase (AT1G08510, homolog GRMZM2G017382) causes a large reduction in saturated fatty acids [92, 93].

### Consistent, inbred specific regulation of processes contributes to recovery gene expression

Among recovering plants, the genotype by temperature effect is significant for 323 genes (Table 1). These genes are enriched for DNA replication, phenylpropanoid metabolism, and other processes (Additional file 10: Table S7). As with cold-grown plants, cold treatment effects conditional on genotype are highly consistent across functionally related genes. In addition, although significant genotype x environment interactions are dominated by genotypes that have similar responses to environmental change and differ only in the magnitude the response [94], processes reveal consistent “crossover” interaction patterns. First, 15 genes are annotated with “DNA conformation change” (Additional file 14: Table S11). For 14 genes, transcript abundances of CG102 plants exposed to cold are lower than CG102 control plants, and transcript abundances of CG60 plants exposed to cold are higher than CG60 control plants (Additional file 14: Table S11). One of these genes Histone H2A (GRMZM2G056231) is shown in Fig. 6c. Second, seven genes are classified within the phenylpropanoid metabolic process. For five of these, CG102 transcript abundance increases in response to cold and CG60 decreases (Additional file 14: Table S11). Four of the genes, *bronze1* (GRMZM2G165390)*, bronze2* (GRMZM2G016241), *anthocyaninless2* (GRMZM2G345717)*,* and *R1 (*GRMZM5G822829) encode proteins known to be necessary for anthocyanin biosynthesis [95]. *R1* (GRMZM5G822829) expression is shown in Fig. 6d. As expected, we noted purpling of CG102 leaves but not CG60 leaves in the recovery period.

## Conclusions

In conclusion, cold temperatures have a major impact on plants in both agricultural and natural settings, and many populations genetically vary for responses to cold and its alleviation. Transcriptome variation is well-known to correlate with plant biochemical and developmental variation. Here, we report a large, core transcriptome response to cold and the alleviation of cold. In cold, these core responses include the down-regulation of photosynthesis and translation-associated transcripts and the up-regulation of transcription factors, kinases, and cytokinin glucoconjugation transcripts. In recovery, these core responses in part indicate preparation for a future stress. Responses include the down-regulation of genes involved in chromatin, lignin, and suberin biosynthesis, and the up-regulation of environmental stress response transcripts, protease inhibitors, lipases, and cuticular wax biosynthesis genes. Genetic diversity of cold response and recovery has two key attributes. First, in response to cold or to its alleviation, the transcript levels of many genes involved in discrete cellular processes such as H_2_O_2_ response, DNA structure, and phenylpropanoid biosynthesis consistently differ between genotypes. Second, the rank of genotypes’ genes’ transcript abundances often changes between control and cold or control and recovery conditions. We speculate that these genotype-specific, discrete, large effect modular responses may be due to genetic differences at single major genes and can be recombined to create novel cold-hardy genotypes.

## Additional files

Additional file 1: **Table S1.** Read numbers and alignment summaries for the 24 RNASeq data sets. (DOCX 19 kb)

Additional file 2: **Table S2.** Tophat alignment summary for sample 1. (DOCX 14 kb)

Additional file 3: **Table S3.** Tophat alignment results from four RNASeq data sets. (DOCX 13 kb)

Additional file 4: **Figure S1.** Frequency of concordant alignments for RNA-Seq read pairs across five parameter sets for four different RNA-Seq datasets. Samples 1 and 2 are CG60. Samples 3 and 4 are CG102. Samples 1 and 3 are from plants harvested 24 h into cold temperature exposure. Samples 2 and 4 are from plants grown only in control conditions. Parameter set details are given in Additional file 2: Table S2. (PDF 4 kb)

Additional file 5: **Table S4.** Number of genes significantly affected by factors at different false discovery rates (FDRs). (DOCX 12 kb)

Additional file 6: **Figure S2.** Molecular function GO terms over-represented (Fisher Test, Benjamini-Hochberg adjusted *p*-value < 0.05) in upregulated (UP) and downregulated (DOWN) genes. D1 COLD-UP and COLD-DOWN refer to genes significantly up or down regulated in cold-grown plants relative to controls; D4 COLD-UP and COLD-DOWN refer to genes significantly up or down regulated in plants that had been exposed to cold relative to controls. CG60-UP refers to transcripts high in inbred CG60 relative to CG102. The *P* values of significantly enriched GO terms with no significant child terms are shown. (PDF 56 kb)

Additional file 7: **Table S5.** A master list of genes expressed within cold and recovery analyses. (CSV 4615 kb)

Additional file 8: **Table S6.** GO terms over-represented amongst genes with significant genotype and treatment main effects in D1 and D4. (XLSX 15 kb)

Additional file 9: **Figure S3.** A plot of the proportion of mesophyll and bundle sheath cell-specific transcripts that are down-regulated in cold. (PDF 4 kb)

Additional file 10: **Table S7.** Significantly enriched biological process, molecular function, and cellular component GO terms amongst genes with significant genotype x environment interactions. (DOCX 13 kb)

Additional file 11: **Table S8.** Transcript abundance estimates of genes from GO category GO:00424542, response to hydrogen peroxide, with significant G x E terms in the analysis of cold-grown plants and their controls (D1). (DOCX 12 kb)

Additional file 12: **Table S9.** GO terms enriched among genes only up-regulated in D4 and among genes up-regulated in both D1 and D4. (DOCX 13 kb)

Additional file 13: **Table S10.** GO terms enriched amongst genes only downregulated in D4 and among genes downregulated in both the cold treatment and recovery treatment relative to controls. (XLSX 12 kb)

Additional file 14: **Table S11.** Genes from two GO categories with significant G x E terms in the analysis of plants with past cold exposure and their controls (D4). (DOCX 13 kb)

## Abbreviations

BS: Bundle sheath
eQTL: Expression quantitative trait locus
FDR: False discovery rate
GO: Gene ontology
M: Mesophyll
MDS: Multidimensional scaling
NDRE: Normalized difference red edge index
PRI: Photochemical reflectance index
RNA-Seq: RNA sequencing
ROS: Reactive oxygen species
