# Supplementary material for: Shared and genetically distinct Zea mays transcriptome responses to ongoing and past low temperature exposure
Source: BMC Genomics. 2018 Oct 20;19:761. doi: 10.1186/s12864-018-5134-7 (PMC6196024; doi:10.1186/s12864-018-5134-7)
Supplement: Supplementary file 1 — Table S1. Read numbers and alignment summaries for the 24 RNASeq data sets. (DOCX 19 kb) [file 12864_2018_5134_MOESM1_ESM.docx]

**Table S1. Read numbers and alignment summaries for the 24 RNASeq data sets.**

| Sample | Description | # Input Read pairs | Percent read pairs with concordant alignment | Percent read pairs with multiple alignments | Percent read pairs with discordant alignment |
| --- | --- | --- | --- | --- | --- |
| 1 | CG60_D1_Stress_R3 | 8,791,849 | 87.30% | 5.30% | 5.00% |
| 2 | CG60_D1_Control_R3 | 9,574,357 | 87.40% | 7.40% | 5.50% |
| 3 | CG102_D1_Stress_R3 | 10,828,940 | 88.60% | 6.20% | 4.40% |
| 4 | CG102_D1_Control_R3 | 9,453,205 | 89.10% | 6.60% | 4.40% |
| 5 | CG60_D4_Stress_R3 | 9606676 | 87.40% | 6% | 5.20% |
| 6 | CG60_D4_Control_R3 | 11,989,454 | 89.00% | 6.60% | 4.20% |
| 7 | CG102_D4_Stress_R3 | 10,677,966 | 88.50% | 6.80% | 4.60% |
| 8 | CG102_D4_Control_R3 | 10,969,420 | 89.60% | 7.20% | 4.20% |
| 9 | CG60_D1_Stress_R2 | 9,752,295 | 88.40% | 5.10% | 4.50% |
| 10 | CG60_D1_Control_R2 | 10,017,674 | 87.70% | 6.80% | 5.30% |
| 11 | CG102_D1_Stress_R2 | 10,150,972 | 88.90% | 5.70% | 4.30% |
| 12 | CG102_D1_Control_R2 | 9,079,336 | 88.90% | 6.40% | 4.40% |
| 13 | CG60_D4_Stress_R2 | 11,170,921 | 84.40% | 17.80% | 9.60% |
| 14 | CG60_D4_Control_R2 | 11,143,742 | 86.50% | 13.80% | 7.50% |
| 15 | CG102_D4_Stress_R2 | 11,111,842 | 89.80% | 6.50% | 3.80% |
| 16 | CG102_D4_Control_R2 | 10,710,871 | 90.40% | 6.90% | 3.50% |
| 17 | CG60_D1_Stress_R1 | 11,118,435 | 89.00% | 5.40% | 4.40% |
| 18 | CG60_D1_Control_R1* | 10,786,956 | 69.20% | 51.10% | 27.80% |
| 19 | CG102_D1_Stress_R1 | 11,794,617 | 90.20% | 5.70% | 3.80% |
| 20 | CG102_D1_Control_R1 | 9,730,063 | 89.70% | 6.30% | 4.10% |
| 21 | CG60_D4_Stress_R1 | 10,677,030 | 87.90% | 7.10% | 5.00% |
| 22 | CG60_D4_Control_R1 | 9,491,372 | 88.80% | 6.80% | 4.20% |
| 23 | CG102_D4_Stress_R1 | 10,045,386 | 89.70% | 6.30% | 4.00% |
| 24 | CG102_D4_Control_R1 | 8,389,239 | 89.40% | 6.30% | 4.30% |

* This sample was excluded from all analyses.
