# Supplementary material for: Shared and genetically distinct Zea mays transcriptome responses to ongoing and past low temperature exposure
Source: BMC Genomics. 2018 Oct 20;19:761. doi: 10.1186/s12864-018-5134-7 (PMC6196024; doi:10.1186/s12864-018-5134-7)
Supplement: Supplementary file 2 — Table S2. Tophat alignment summary for sample 1. (DOCX 14 kb) [file 12864_2018_5134_MOESM2_ESM.docx]

| **Test** | **Maximum read mismatches** | **Maximum read gap length** | **Maximum edit distance** | **Reads with unique concordant**  **alignment** | **Reads with multiple alignment** | **Reads with discordant alignment** | **Sites different than reference in Chr1** | **Variable nucleotides in Chr1** |
| --- | --- | --- | --- | --- | --- | --- | --- | --- |
| 1 | 2 | 2 | 2 | 71.50% | 5.10% | 2.60% | 10,263 | 445 |
| 2 | 6 | 6 | 6 | 81.30% | 5.30% | 3.50% | 19,211 | 1,329 |
| 3 | 8 | 8 | 8 | 83.00% | 5.30% | 3.90% | 21,465 | 1,603 |
| 4 | 10 | 10 | 10 | 84.20% | 5.40% | 4.10% | 23,086 | 1,780 |
| 5 | 12 | 12 | 12 | 85.10% | 5.40% | 4.30% | 24,270 | 1,922 |
| 6 | 14 | 14 | 14 | 85.80% | 5.30% | 4.50% | 25,516 | 2,118 |
| 7 | 16 | 16 | 16 | 86.40% | 5.30% | 4.70% | 26,343 | 2,145 |
| 8 | 18 | 18 | 18 | 86.90% | 5.30% | 4.90% | 27,307 | 2,290 |
| 9 | 20 | 20 | 20 | 87.30% | 5.30% | 5.00% | 27,955 | 2,229 |
| 10 | 22 | 22 | 22 | 87.90% | 5.40% | 5.30% | 29,116 | 2,477 |
| 11 | 24 | 24 | 24 | 88.20% | 5.40% | 5.40% | 29,604 | 2,489 |
| 12 | 26 | 26 | 26 | 88.40% | 5.40% | 5.60% | 30,128 | 2,585 |
| 13 | 28 | 28 | 28 | 88.70% | 5.40% | 5.80% | 30,698 | 2,556 |
| 14 | 30 | 30 | 30 | 89.10% | 5.40% | 5.90% | 31,323 | 2,556 |
| 15 | 32 | 32 | 32 | 89.30% | 5.40% | 6.00% | 31,711 | 2,658 |
| 16 | 34 | 34 | 34 | 89.20% | 5.40% | 6.10% | 31,965 | 2,635 |
| 17 | 36 | 36 | 36 | 89.10% | 5.50% | 6.20% | 32,100 | 2,630 |

**Table S2.** **Tophat alignment summary for sample 1.** Alignment results from using different parameter values for –read-mismatches -- read-gap-length, and –read-edit-distance. The column “variable nucleotides in Chr1” gives the number of genomic positions at which aligned reads have different nucleotides. Reads that satisfy test criteria set nine or better are used in this study.
