# Supplementary material for: Shared and genetically distinct Zea mays transcriptome responses to ongoing and past low temperature exposure
Source: BMC Genomics. 2018 Oct 20;19:761. doi: 10.1186/s12864-018-5134-7 (PMC6196024; doi:10.1186/s12864-018-5134-7)
Supplement: Supplementary file 3 — Table S3. Tophat alignment results from four RNASeq data sets. (DOCX 13 kb) [file 12864_2018_5134_MOESM3_ESM.docx]

| **sample** | **Parameter**  **set** | **Allowed mismatches** | **allowed**  **in/del** | **Reads with unique concordant**  **alignment** | **Reads with multiple alignment** | **Reads with discordant alignment** |
| --- | --- | --- | --- | --- | --- | --- |
| 1 | 1 | 2 | 3 | 71.50% | 5.10% | 2.60% |
| 2 |  |  |  | 72.60% | 7.10% | 3.40% |
| 3 |  |  |  | 74.90% | 6.20% | 2.30% |
| 4 |  |  |  | 75.90% | 6.70% | 2.50% |
| 1 | 5 | 12 | 15 | 85.10% | 5.40% | 4.30% |
| 2 |  |  |  | 85.80% | 7.40% | 4.80% |
| 3 |  |  |  | 86.90% | 6.20% | 3.80% |
| 4 |  |  |  | 87.60% | 6.60% | 3.80% |
| 1 | 9 | 20 | 23 | 87.30% | 5.30% | 5.00% |
| 2 |  |  |  | 87.40% | 7.40% | 5.50% |
| 3 |  |  |  | 88.60% | 6.20% | 4.40% |
| 4 |  |  |  | 89.10% | 6.60% | 4.40% |
| 1 | 13 | 28 | 31 | 88.70% | 5.40% | 5.80% |
| 2 |  |  |  | 88.70% | 7.40% | 6.20% |
| 3 |  |  |  | 89.70% | 6.20% | 5.00% |
| 4 |  |  |  | 90.10% | 6.80% | 5.00% |
| 1 | 17 | 36 | 39 | 89.10% | 5.50% | 6.20% |
| 2 |  |  |  | 88.90% | 7.40% | 6.60% |
| 3 |  |  |  | 89.90% | 6.30% | 5.40% |
| 4 |  |  |  | 90.30% | 6.80% | 5.30% |

**Table S3. Tophat alignment results from four RNASeq data sets.** Samples 1 to 4 (CG60_D1_Stress, CG60_D1_Control, CG102_D1_Stress and CG102_D1_Control from replicate 3) were aligned with Tophat using four parameter sets.
