# Supplementary figures and images for: Shared and genetically distinct Zea mays transcriptome responses to ongoing and past low temperature exposure
Source: BMC Genomics. 2018 Oct 20;19:761. doi: 10.1186/s12864-018-5134-7 (PMC6196024; doi:10.1186/s12864-018-5134-7)

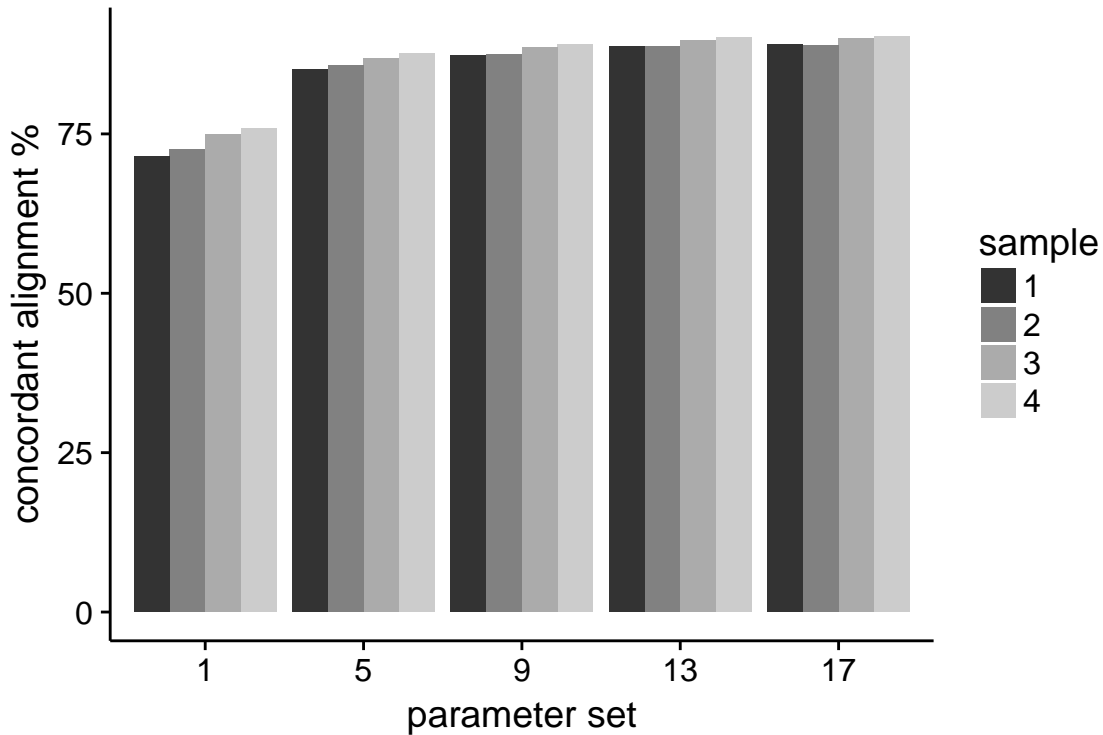

Supplement: Supplementary file 4 — Figure S1. Frequency of concordant alignments for RNA-Seq read pairs across five parameter sets for four different RNA-Seq datasets. Samples 1 and 2 are CG60. Samples 3 and 4 are CG102. Samples 1 and 3 are from plants harvested 24 h into cold temperature exposure. Samples 2 and 4 are from plants grown only in control conditions. Parameter set details are given in Additional file 2: Table S2. (PDF 4 kb) [file 12864_2018_5134_MOESM4_ESM.pdf]

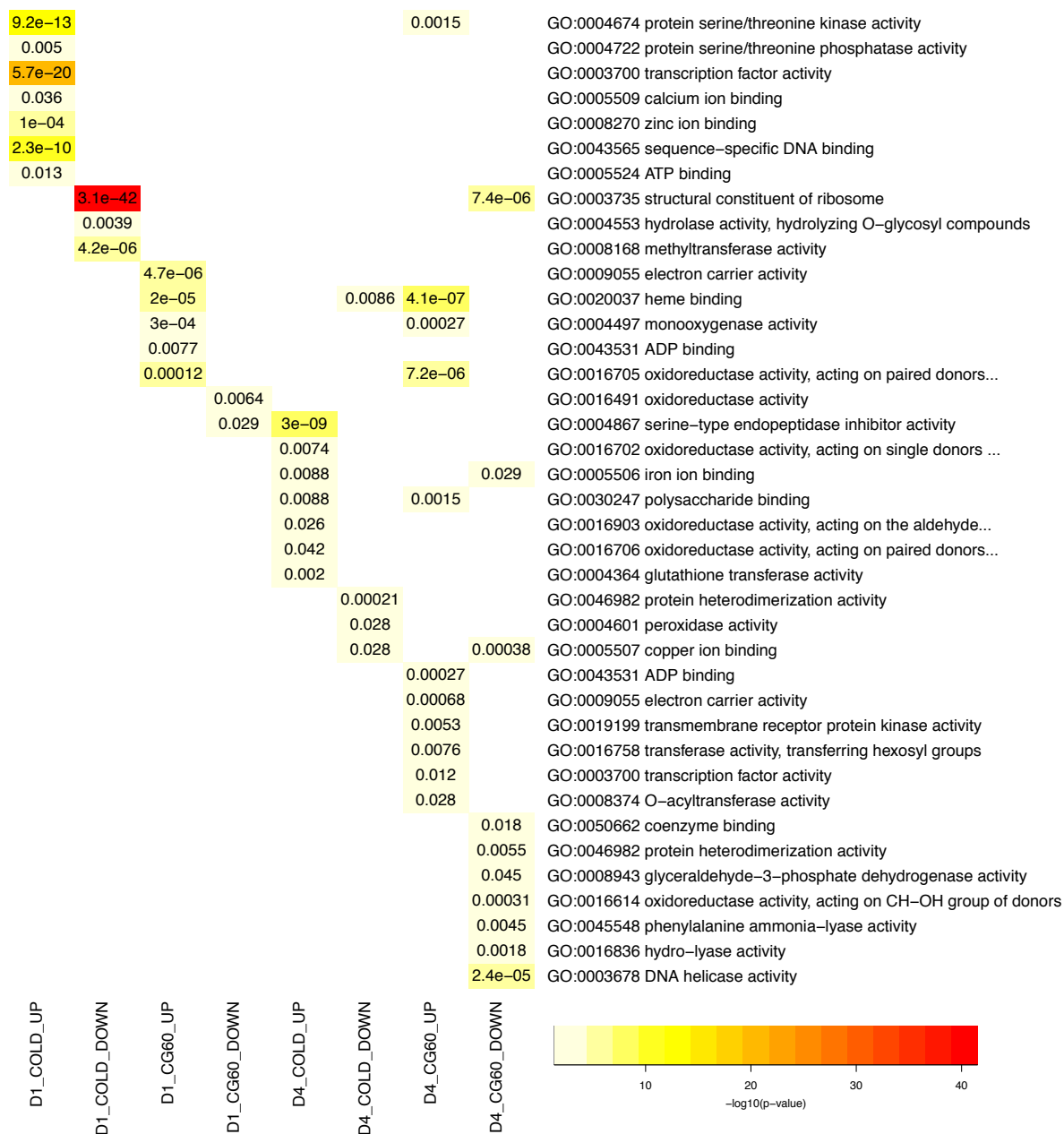

Supplement: Supplementary file 6 — Figure S2. Molecular function GO terms over-represented (Fisher Test, Benjamini-Hochberg adjusted p-value < 0.05) in upregulated (UP) and downregulated (DOWN) genes. D1 COLD-UP and COLD-DOWN refer to genes significantly up or down regulated in cold-grown plants relative to controls; D4 COLD-UP and COLD-DOWN refer to genes significantly up or down regulated in plants that had been exposed to cold relative to controls. CG60-UP refers to transcripts high in inbred CG60 relative to CG102. The P values of significantly enriched GO terms with no significant child terms are shown. (PDF 56 kb) [file 12864_2018_5134_MOESM6_ESM.pdf]

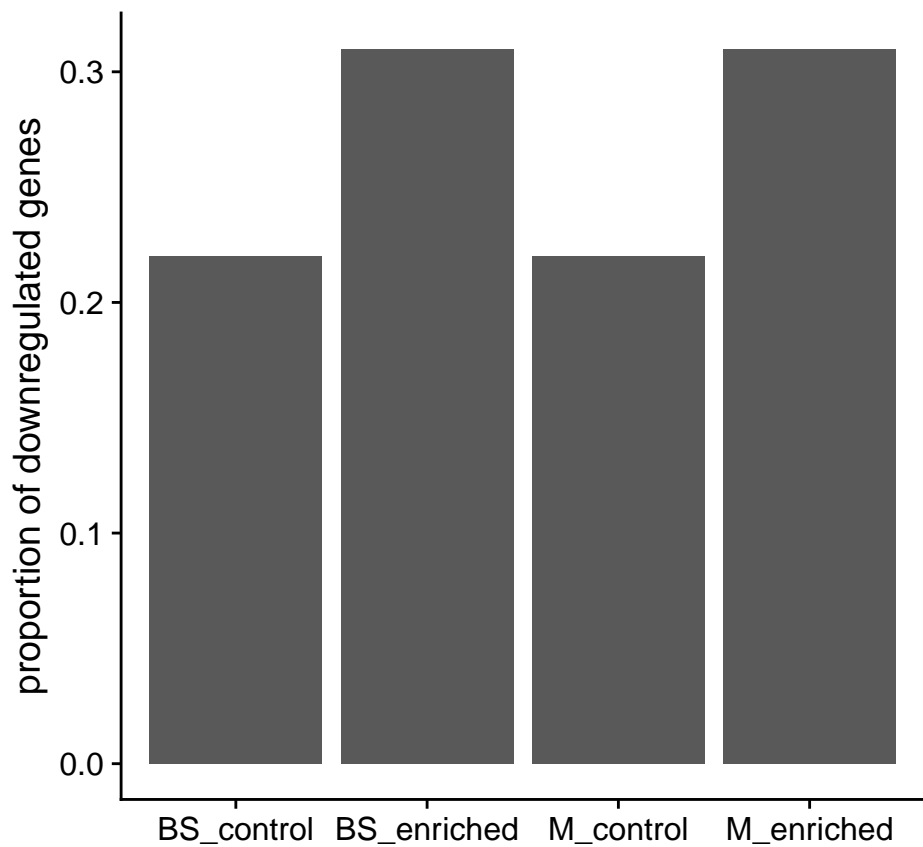

Supplement: Supplementary file 9 — Figure S3. A plot of the proportion of mesophyll and bundle sheath cell-specific transcripts that are down-regulated in cold. (PDF 4 kb) [file 12864_2018_5134_MOESM9_ESM.pdf]
