# Supplementary material for: Shared and genetically distinct Zea mays transcriptome responses to ongoing and past low temperature exposure
Source: BMC Genomics. 2018 Oct 20;19:761. doi: 10.1186/s12864-018-5134-7 (PMC6196024; doi:10.1186/s12864-018-5134-7)
Supplement: Supplementary file 5 — Table S4. Number of genes significantly affected by factors at different false discovery rates (FDRs). (DOCX 12 kb) [file 12864_2018_5134_MOESM5_ESM.docx]

| **FDR** | **time point*** | **treatment** | **genotype** | **interaction** |
| --- | --- | --- | --- | --- |
| 5% | D1 | 10,549 | 6,514 | 1,541 |
|  | D4 | 556 | 7,694 | 323 |
| 1% | D1 | 8,165 | 4,842 | 837 |
|  | D4 | 336 | 5,794 | 155 |
| 0.1% | D1 | 6,037 | 3,630 | 415 |
|  | D4 | 199 | 4,371 | 66 |

**Table S4. Number of genes significantly affected by factors at different false discovery rates (FDRs).**

*D1 refers to RNA-Seq data from samples grown in cold and their controls. D4 refers to RNA-Seq data from samples 24h after the cold treatment and their controls. After filtering out low expressed genes from the 39,469 identified genes, the transcript abundances of 22,942 and 22,867 genes were modelled in D1 and D4, respectively.
