# Supplementary material for: Shared and genetically distinct Zea mays transcriptome responses to ongoing and past low temperature exposure
Source: BMC Genomics. 2018 Oct 20;19:761. doi: 10.1186/s12864-018-5134-7 (PMC6196024; doi:10.1186/s12864-018-5134-7)
Supplement: Supplementary file 10 — Table S7. Significantly enriched biological process, molecular function, and cellular component GO terms amongst genes with significant genotype x environment interactions. (DOCX 13 kb) [file 12864_2018_5134_MOESM10_ESM.docx]

**Table S7.** Significantly enriched biological process, molecular function, and cellular component GO terms amongst genes with significant genotype x environment interactions. Interaction P values are FDR corrected and given if P<0.05.

| **GO_acc** | **Term_type** | **Term** | **Stress timepoint (D1) interaction** | **Recovery timepoint (D4) interaction** |
| --- | --- | --- | --- | --- |
| GO:0030528 | F | transcription regulator activity | 0.029 |  |
| GO:0003700 | F | transcription factor activity | 0.025 |  |
| GO:0004497 | F | monooxygenase activity | 0.004 |  |
| GO:0016705 | F | oxidoreductase activity, acting on paired donors, with incorporation or reduction of molecular oxygen | 9.70E-05 |  |
| GO:0005506 | F | iron ion binding | 1.90E-05 |  |
| GO:0020037 | F | heme binding | 9.70E-05 |  |
| GO:0016491 | F | oxidoreductase activity | 0.01 |  |
| GO:0009055 | F | electron carrier activity | 0.0039 |  |
| GO:0046906 | F | tetrapyrrole binding | 0.00014 |  |
| GO:0004867 | F | serine-type endopeptidase inhibitor activity | 0.05 |  |
| GO:0003678 | F | DNA helicase activity |  | 7.20E-05 |
| GO:0055114 | P | oxidation reduction | 0.037 |  |
| GO:0042542 | P | response to hydrogen peroxide | 0.021 |  |
| GO:0009408 | P | response to heat | 0.021 |  |
| GO:0071103 | P | DNA conformation change |  | 6.00E-07 |
| GO:0006270 | P | DNA replication initiation |  | 1.10E-07 |
| GO:0006260 | P | DNA replication |  | 0.0027 |
| GO:0032392 | P | DNA geometric change |  | 1.60E-06 |
| GO:0032508 | P | DNA duplex unwinding |  | 1.60E-06 |
| GO:0006261 | P | DNA-dependent DNA replication |  | 1.60E-05 |
| GO:0006259 | P | DNA metabolic process |  | 0.053 |
| GO:0009698 | P | phenylpropanoid metabolic process |  | 0.07 |
| GO:0032993 | C | protein-DNA complex |  | 0.017 |
| GO:0000785 | C | chromatin |  | 1.60E-06 |
| GO:0042555 | C | MCM complex |  | 1.30E-08 |
| GO:0005694 | C | chromosome |  | 1.70E-06 |
| GO:0044454 | C | nuclear chromosome part |  | 6.40E-06 |
| GO:0000228 | C | nuclear chromosome |  | 2.80E-05 |
