# Supplementary material for: Shared and genetically distinct Zea mays transcriptome responses to ongoing and past low temperature exposure
Source: BMC Genomics. 2018 Oct 20;19:761. doi: 10.1186/s12864-018-5134-7 (PMC6196024; doi:10.1186/s12864-018-5134-7)
Supplement: Supplementary file 11 — Table S8. Transcript abundance estimates of genes from GO category GO:00424542, response to hydrogen peroxide, with significant G x E terms in the analysis of cold-grown plants and their controls (D1). (DOCX 12 kb) [file 12864_2018_5134_MOESM11_ESM.docx]

**Table S8:** Transcript abundance estimates of genes from GO category GO:00424542, response to hydrogen peroxide, with significant G x E terms in the analysis of cold-grown plants and their controls (D1). Similar genotypic x environment interaction patterns among genes are noted.

| Number | gene_ID | 1_Control_102 | 1_Stress_102 | 1_Control_60 | 1_Stress_60 | Stress_CG60 >Stress_CG102 |
| --- | --- | --- | --- | --- | --- | --- |
| 65 | GRMZM2G162688 | 37.22 | 34.52 | 30.68 | 122.43 | 1 |
| 166 | GRMZM2G165919 | 22.08 | 63.12 | 18.12 | 13.50 |  |
| 214 | GRMZM2G428391 | 142.69 | 1138.02 | 141.50 | 2754.02 | 2 |
| 377 | GRMZM2G109071 | 43.51 | 24.67 | 23.85 | 13.87 |  |
| 472 | GRMZM2G155242 | 41.07 | 449.85 | 43.40 | 1028.46 | 3 |
| 513 | GRMZM2G325575 | 519.88 | 420.70 | 1062.00 | 364.71 |  |
| 648 | GRMZM2G400470 | 16.23 | 21.14 | 15.00 | 45.08 | 4 |
| 752 | GRMZM2G071630 | 264.89 | 237.23 | 109.64 | 282.30 | 5 |
| 766 | GRMZM2G034157 | 3.61 | 6.05 | 0.74 | 4.41 | 6 |
| 805 | GRMZM2G078465 | 1.91 | 261.37 | 1.90 | 86.09 |  |
| 876 | GRMZM2G004161 | 17.23 | 41.30 | 26.91 | 30.36 |  |
| 914 | GRMZM2G024718 | 2.39 | 1.76 | 4.15 | 9.84 | 7 |
| 1286 | GRMZM2G070863 | 8.35 | 21.92 | 6.81 | 30.68 | 8 |
| 1371 | GRMZM2G121715 | 10.95 | 9.46 | 1.65 | 0.55 |  |
| 1522 | GRMZM2G176307 | 89.16 | 64.15 | 41.29 | 81.06 | 9 |
