# Supplementary material for: Shared and genetically distinct Zea mays transcriptome responses to ongoing and past low temperature exposure
Source: BMC Genomics. 2018 Oct 20;19:761. doi: 10.1186/s12864-018-5134-7 (PMC6196024; doi:10.1186/s12864-018-5134-7)
Supplement: Supplementary file 12 — Table S9. GO terms enriched among genes only up-regulated in D4 and among genes up-regulated in both D1 and D4. (DOCX 13 kb) [file 12864_2018_5134_MOESM12_ESM.docx]

**Table S9.** GO terms enriched among genes only up-regulated in D4 and among genes up-regulated in both D1 and D4.

| Up-regulated in D1_and/or D4 | GO term | Ontology | Description | Number in input list | Number in BG/Ref | P-value | FDR |
| --- | --- | --- | --- | --- | --- | --- | --- |
| D1_and_D4 | GO:0042221 | P | response to chemical stimulus | 33 | 2052 | 9.50E-11 | 5.50E-08 |
| D1_and_D4 | GO:0050896 | P | response to stimulus | 42 | 3551 | 1.60E-09 | 4.70E-07 |
| D1_and_D4 | GO:0006950 | P | response to stress | 32 | 2243 | 3.70E-09 | 7.20E-07 |
| D1_and_D4 | GO:0009628 | P | response to abiotic stimulus | 24 | 1397 | 1.70E-08 | 2.50E-06 |
| D1_and_D4 | GO:0009415 | P | response to water | 11 | 264 | 4.90E-08 | 5.70E-06 |
| D1_and_D4 | GO:0009642 | P | response to light intensity | 7 | 101 | 6.00E-07 | 5.80E-05 |
| D1_and_D4 | GO:0006970 | P | response to osmotic stress | 12 | 538 | 7.30E-06 | 0.0006 |
| D1_and_D4 | GO:0009266 | P | response to temperature stimulus | 11 | 464 | 1.00E-05 | 0.00074 |
| D1_and_D4 | GO:0055114 | P | oxidation reduction | 23 | 1931 | 1.80E-05 | 0.00093 |
| D1_and_D4 | GO:0009651 | P | response to salt stress | 11 | 491 | 1.70E-05 | 0.00093 |
| D1_and_D4 | GO:0009644 | P | response to high light intensity | 5 | 64 | 1.50E-05 | 0.00093 |
| D1_and_D4 | GO:0009414 | P | response to water deprivation | 8 | 254 | 2.50E-05 | 0.0012 |
| D1_and_D4 | GO:0010035 | P | response to inorganic substance | 12 | 645 | 4.20E-05 | 0.0019 |
| D1_and_D4 | GO:0006979 | P | response to oxidative stress | 9 | 391 | 8.50E-05 | 0.0035 |
| D1_and_D4 | GO:0009409 | P | response to cold | 7 | 318 | 0.00069 | 0.026 |
| D1_and_D4 | GO:0009408 | P | response to heat | 5 | 163 | 0.001 | 0.035 |
| D1_and_D4 | GO:0006790 | P | sulfur metabolic process | 5 | 164 | 0.001 | 0.035 |
| D1_and_D4 | GO:0016491 | F | oxidoreductase activity | 24 | 2055 | 1.60E-05 | 0.0023 |
| D1_and_D4 | GO:0016765 | F | "transferase activity, transferring alkyl or aryl (other than methyl) groups" | 5 | 126 | 0.00032 | 0.024 |
| D1_and_D4 | GO:0003824 | F | catalytic activity | 70 | 11519 | 0.00065 | 0.032 |
| D1_and_D4 | GO:0005576 | C | extracellular region | 10 | 575 | 0.00032 | 0.037 |
| D4_only | GO:0006629 | P | lipid metabolic process | 11 | 1068 | 7.30E-05 | 0.011 |
| D4_only | GO:0004866 | F | endopeptidase inhibitor activity | 8 | 143 | 4.30E-09 | 3.90E-07 |
| D4_only | GO:0030414 | F | peptidase inhibitor activity | 8 | 143 | 4.30E-09 | 3.90E-07 |
| D4_only | GO:0004857 | F | enzyme inhibitor activity | 8 | 236 | 1.80E-07 | 1.10E-05 |
| D4_only | GO:0004867 | F | serine-type endopeptidase inhibitor activity | 6 | 116 | 6.20E-07 | 2.80E-05 |
| D4_only | GO:0030234 | F | enzyme regulator activity | 8 | 552 | 7.70E-05 | 0.0028 |
| D4_only | GO:0005506 | F | iron ion binding | 10 | 1187 | 0.00076 | 0.023 |
| D4_only | GO:0016491 | F | oxidoreductase activity | 18 | 3266 | 0.001 | 0.027 |
