# Supplementary material for: Shared and genetically distinct Zea mays transcriptome responses to ongoing and past low temperature exposure
Source: BMC Genomics. 2018 Oct 20;19:761. doi: 10.1186/s12864-018-5134-7 (PMC6196024; doi:10.1186/s12864-018-5134-7)
Supplement: Supplementary file 14 — Table S11. Genes from two GO categories with significant G x E terms in the analysis of plants with past cold exposure and their controls (D4). (DOCX 13 kb) [file 12864_2018_5134_MOESM14_ESM.docx]

**Table S11.** Genes from two GO categories with significant G x E terms in the analysis of plants with past cold exposure and their controls (D4). Similar genotypic x environment interaction patterns among genes are noted. GO:0009698 is phenylpropanoid metabolic process. GO:0071103 is DNA conformation change.

| GO_term | Gene_Name | CG102_control | CG60_control | CG102_cold | CG60_cold | Cold CG60 > control CG60 and cold CG102< control CG102 | Cold CG102 > control C102 and cold CG60< control CG60 |
| --- | --- | --- | --- | --- | --- | --- | --- |
| GO:0071103 | GRMZM2G066101 | 14.30 | 5.80 | 12.52 | 15.27 | yes |  |
| GO:0071103 | GRMZM2G056231 | 5.97 | 1.59 | 3.04 | 2.97 | yes |  |
| GO:0071103 | GRMZM2G472696 | 15.56 | 7.12 | 10.45 | 11.35 | yes |  |
| GO:0071103 | GRMZM2G139894 | 20.68 | 3.52 | 17.15 | 8.81 | yes |  |
| GO:0071103 | GRMZM2G450055 | 4.89 | 3.13 | 4.64 | 8.05 | yes |  |
| GO:0071103 | GRMZM2G479684 | 15.82 | 4.26 | 7.92 | 4.77 | yes |  |
| GO:0071103 | GRMZM2G112074 | 3.86 | 1.15 | 3.51 | 3.62 | yes |  |
| GO:0071103 | GRMZM2G461447 | 1.41 | 0.16 | 0.71 | 0.83 | yes |  |
| GO:0071103 | GRMZM2G075978 | 18.75 | 4.41 | 17.64 | 9.97 | yes |  |
| GO:0071103 | GRMZM2G162445 | 10.55 | 4.56 | 9.89 | 10.78 | yes |  |
| GO:0071103 | GRMZM2G095865 | 85.87 | 29.04 | 68.31 | 57.84 | yes |  |
| GO:0071103 | AC203761.3_FG002 | 22.01 | 8.61 | 18.20 | 19.38 | yes |  |
| GO:0071103 | GRMZM2G100639 | 12.09 | 3.59 | 11.44 | 7.73 | yes |  |
| GO:0071103 | GRMZM2G342515 | 10.81 | 2.37 | 5.73 | 3.11 | yes |  |
| GO:0071103 | GRMZM2G305046 | 78.52 | 24.32 | 42.41 | 24.00 | no |  |
| GO:0009698 | GRMZM5G822829 | 24.09 | 32.53 | 47.41 | 11.52 |  | yes |
| GO:0009698 | GRMZM2G097706 | 0.73 | 7.78 | 3.40 | 3.77 |  | yes |
| GO:0009698 | GRMZM2G345717 | 28.32 | 15.42 | 74.16 | 10.17 |  | yes |
| GO:0009698 | GRMZM2G016241 | 38.01 | 29.83 | 77.22 | 16.17 |  | yes |
| GO:0009698 | GRMZM2G165390 | 10.86 | 9.33 | 22.01 | 5.32 |  | yes |
| GO:0009698 | GRMZM2G367668 | 4.69 | 2.59 | 1.82 | 3.11 |  | no |
| GO:0009698 | GRMZM2G034360 | 67.23 | 53.69 | 48.05 | 42.84 |  | no |
